# Botulinum toxin therapy: past, present and future developments

**DOI:** 10.1007/s00702-022-02494-5

**Published:** 2022-04-09

**Authors:** Dirk Dressler, Eric A. Johnson

**Affiliations:** 1 Movement Disorders Section, Department of Neurology, Hannover Medical School, Carl-Neuberg-Str. 1, 30625 Hannover, Germany; 2 Botulinum Toxins Laboratory, Department of Bacteriology, University of Wisconsin, Madison, WI USA

**Keywords:** Botulinum toxin, Therapy, Drugs, Indications, Future developments, Manufacturers

## Abstract

Although botulinum toxin (BT) is now being used in a large number of different indications in numerous medical specialties, there is still dynamic and rapid development. Treatment algorithms were improved by the introduction of BT short-interval therapy, BT high-dose therapy and improved dosing guidelines. Ultrasound guidance may be helpful in special situations. New indication areas including depression and inflammatory processes are being explored. Drug development projects are mainly focusing on onabotulinumtoxinA analogues, some are addressing liquid preparations and modifications of BT's duration of action. Recombinant BT may simplify production processes. Cell-based assays for potency measurement will soon be required by registration authorities. Treatment algorithms will be further refined and indications will be expanded. New indication areas are still uncertain. BT type A will remain the drug substance of choice. Removal of complexing proteins seems logical. Whether there is a need for BT drugs with modified duration of action and for liquid preparations, is unclear. Bringing BT therapy to those who need it, is the biggest challenge. Current high-price business models need to be changed, either by employing a biosimilar registration approach or by referring to companies from countries where business models are based on different cost structures.

## Introduction

Botulinum toxin (BT) has undergone one of the most remarkable transitions in the history of mankind: once infamous as a food safety hazard and a means of biological warfare, it is now a highly effective drug for an amazing number of different disorders. For many of them, BT has revolutionized their therapy. Although BT drugs and their therapeutic applications have come a long way to where they are now, there is still dynamic and rapid development. We want to review this development in BT's therapeutic applications and its drug development. In an outlook, we want to sketch some future development perspectives.

## Therapeutic applications

BT produces a well-controllable fully reversible blockade of cholinergic synapses. This has generated two main indication areas. Injected into muscle tissue, BT produces neuromuscular blockade with muscle relaxation, which may be used to treat muscle hyperactivity disorders such as various forms of dystonia, spasticity, infantile cerebral palsy, hemifacial spasm, tics, tremor and motility disorders of the bladder and the gastrointestinal tract (Dressler 2012). This indication area currently covers around 26 indications in some six medical specialties. Of those, blepharospasm, cervical dystonia, post stroke spasticity, infantile cerebral palsy, hemifacial spasm, detrusor overactivity and strabismus are registered indications. Besides, the use of BT in esthetic medicine is also based on this muscle relaxing effect, which may reduce wrinkles generated by underlying muscle hyperactivity.

If BT is injected into exocrine gland tissues, it reduces the production of sweat, saliva and tears, thus offering elegant treatment of hyperhidrosis, sialorrhea and crocodile's tear phenomenon (Dressler 2012). Hyperhidrosis and sialorrhea, the most common conditions in this indication area, are registered indications.

The use of BT to treat chronic migraine is a more recently approved indication (Aurora et al. 2010). It opens up a different indication area. However, the pharmacological and physiological basis of this effect remains unclear, but certainly reaches beyond classic cholinergic effects.

*Treatment algorithms:* BT therapy was recently advanced by three major modifications of the treatment algorithms. Originally, BT therapy was applied with fixed interinjection intervals of at least 12 weeks. This recommendation was generated, after short interinjection intervals were identified as a risk factor for BT antibody formation. When it became apparent, that these fixed intervals did not match the duration of the therapeutic effect in a substantial number of dystonia patients (Dressler et al. 2015a), BT applications with reduced interinjection intervals were tested. After these injections did not produce BT antibody formation, BT short-interval therapy was formulated (Dressler and Adib Saberi 2017). Short interval therapy can substantially prolong the duration of the treatment effect in a large number of patients, thus improving their quality of life considerably. So far, short interval BT therapy was only described for incobotulinumtoxinA (Xeomin^®^, Merz Pharmaceuticals, Frankfurt/M), which is believed to excel with substantially reduced antigenicity due to an improved specific biological activity and a lack of complexing proteins (Dressler and Hallett 2006; Dressler and Bigalke 2017). Further studies will have to test, whether also other BT drugs may be used for this purpose.

Originally, it was also recommended that BT total doses should not exceed around 400MU per injection series to reduce risks of BT antibody formation and systemic adverse effects. In a pivotal study (Dressler et al. 2015b) it was shown, that—wherever necessary—total BT doses may, indeed, exceed 400MU without risks of BT antibody formation and systemic adverse effects. In this study, the average total dose was 570.1 ± 158.9MU and in singular patients it reached 1200MU. These findings established BT high-dose therapy. A subsequent study (Wissel et al. 2017) using total doses 800MU confirmed the original study. BT high-dose therapy allows generation of more robust therapeutic effects and treatment of more widespread conditions, especially in dystonia and in spasticity. For the treatment of dystonia, this redefines the dividing line between the use of BT therapy and deep brain stimulation (Dresssler et al. 2016). Again, both studies introducing BT high-dose therapy were using incobotulinumtoxinA. Other BT drugs, therefore, also need to be tested for this purpose.

For motor indications, BT therapy crucially depends on the development of an optimal injection scheme describing the target muscle selection and their BT dosing. These injection schemes are highly individual and vary from patient to patient to a large extent. So far, they were based entirely on expert opinion. Recently, statistical analyses of the large digital data base of a special reference center provided exact data on typical BT doses, dose variabilities and dosing limits for all relevant target muscles used for treatment of dystonia and spasticity (Dressler et al. 2021a). This analysis also provided an overview over total BT doses and target selections for all major clinical presentations of dystonia and spasticity (Dressler et al. 2021a).

*Guided BT application*: Originally, BT application to the body was performed using anatomical hallmarks only. Soon later, electromyography with recording of the muscle hyperactivity or stimulation of the target muscle directly through a special injection cannula were introduced to increase application precision. After some time, ultrasound imaging was suggested to visualize the target tissue and its adjacent neural and vascular structures. It was hypothesized, that this could increase therapeutic efficacy and reduce adverse effects. Although this hypothesis was never proven, it seems reasonable to use the increased application precision provided by ultrasound guidance in forearm muscles in very focal dystonia forms such as writer's cramp and musician's cramp, in target muscles positioned deep within the body and in children, where anatomical hallmarks are sparse (Walter and Dressler 2014).

*New indications*: Within the pain indication area, various indications other than chronic migraine are currently being studied (Siongco et al. 2020). However, no new registration studies are currently being performed.

*New indication areas:* Several years ago, it was demonstrated that BT injections into the forehead might reduce depression (Finzi and Wasserman 2006, Wollmer et al. 2012). Four subsequent randomized controlled trials confirmed these anti-depressive effects (for review, see Kruger and Wollmer 2015; Qian et al. 2020), whilst the fifth one intended for eventual drug registration failed to produce significant therapeutic effects (Brin et al. 2020. Even more so than with pain indications, mechanisms underlying BT effects on depression are unknown. Another potentially interesting indication area for BT therapy could be the modification of inflammatory reactions as originally suggested several years ago (Dykstra et al. 2007). Several subsequent studies suggest therapeutic effects after intra-articular BT injections. Registration studies are missing. Intradermal BT injections are usually ineffective for articular pain reduction (Voller et al. 2003).

## Drug development

**Fig. 1:**
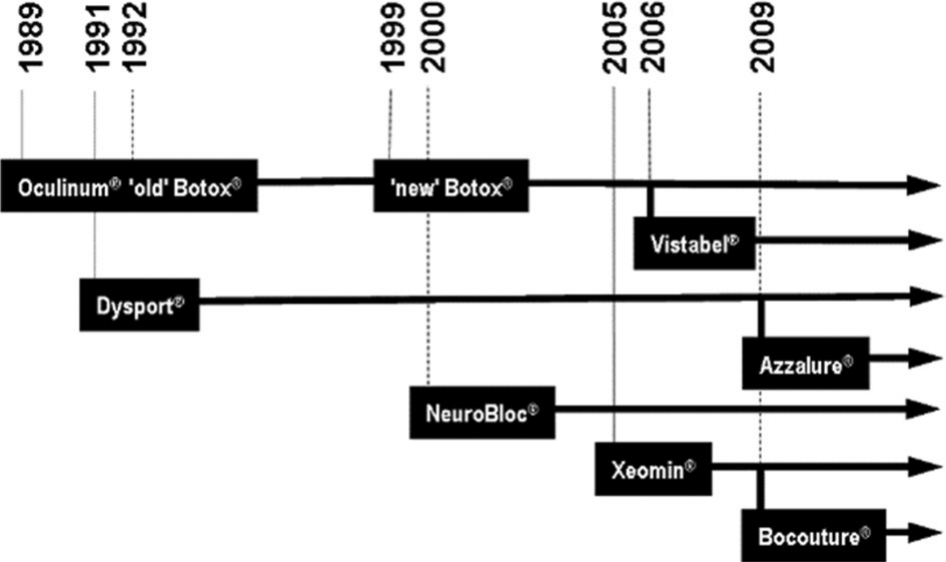
The genealogy of botulinum toxin drugs. From: Dressler (2012)

Figure 1 shows the genealogy of the main BT drugs registered so far. Milestones of drug development were the first registration of a BT drug in 1989, the first registration of a BT type B drug—also being the first drug in a liquid preparation—in 2000 and the first registration of a BT drug without complexing proteins in 2005 (Dressler 2012). A recent analysis revealed that in China as early as 1997 another BT type A drug (lanbotulinumtoxinA, Lantox^®^, Lanzhou Institute of Biological Products, Lanzhou, Gansu Province, China) had been registered (Dressler et al. 2021b). It has similar properties compared to onabotulinumtoxinA.

Table 1 shows an overview about ongoing BT drug development projects (Dressler 2021). Most of the projects are aiming at developing onabotulinumtoxinA analogues, mainly for esthetic purposes. The Masport^®^ development project targets abobotulinumtoxinA, the Innotox^®^ development project targets incobotulinumtoxinA. A liquid preparation BT drug has been available for many years (Setler 2000). New projects are trying to develop new liquid preparations or to manufacture liquid formulations of existing BT drugs. One development project (daxibotulinumtoxinA, Revance, Nashville, TN, USA) (Carruthers et al. 2017) is based on the combination of BT type A with a proprietary protein. Originally, it was claimed that this protein would allow facilitate BT absorption, so that injections for application would become obsolete. After that claim could not be substantiated, it was claimed, that the proprietary protein could prolong and BT's duration of action and reduce its adverse effects. Again, this claim could not be substantiated. Currently. the company tries to market their proprietary protein as a substitute for human serum albumin, which is widely used in BT drugs and other pharmaceuticals and never generated any safety problems. Coretox^®^, a BT type A toxin drug without complexing proteins, also stresses its lack of biological excipients including human serum albumin.

**Table 1:** Therapeutic botulinum toxin preparations

Generic name	Trade name	Manufacturers and partners (past and present)	Country	Specifics
OnabotulinumtoxinA	Botox Botox^®^ Cosmetics^®^ Vistabel^®^	Allergan-AbbVie	USA/Ireland	BT-A
AbobotulinumtoxinA	Dysport^®^ Azzalure^®^ Reloxin^®^	Ipsen/Medicis	UK/France/USA	BT-A
IncobotulinumtoxinA	Xeomin^®^ Xeomin Cosmetics^®^ Bocouture^®^	Merz Pharmaceuticals	Germany	BT-A, no complexing proteins
RimabotulinumtoxinB	NeuroBloc^®^ Myobloc^®^ Nerbloc^®^	US WorldMeds/ Eisai/Sloan/Elan/Solstice	USA	BT-B, liquid preparation
LanbotulinumtoxinA	Hengli^®^ Lantox^®^ Lanzox^®^ CBTX-A^®^ Prosigne^®^ Redux^®^ Liftox^®^ Dituroxal^®^	Lanzhou Institute of Biological Products/Hugh Source	P.R. China	BT-A, Botox^®^ analogon
	Neuronox^®^ Meditoxin^®^ Botulift^®^ Cunox^®^	Medytox	R. Korea	BT-A, Botox^®^ analogon
	Coretox^®^	Medytox	R. Korea	BT-A, Xeomin^®^ analogon, no complexing proteins, no biological excipients
	Innotox^®^	Medytox/Allergan	R. Korea/USA	BT-A, liquid preparation
	Botulax^®^ Zentox^®^ Regenox^®^	Hugel	R. Korea	
PrabotulinumtoxinA	Nabota^®^ Jeuveau^®^ Evosyal^®^	Daewong/Evolus-Alphaeon	R. Korea/USA	BT-A, Botox^®^ analogon
DaxibotulinumtoxinA	RTT150	Revance	USA	BT-A, protein additive
		Revance/Mylan	USA/Netherlands	BT-A, Botox^®^ analogon, biosimilar approach
	Relatox^®^	Microgen	Russia	BT-A, Botox^®^ analogon
	Botulax^®^	Hugel	R. Korea	BT-A, Botox^®^ analogon
	Masport^®^	Masoundarou	I.R. Iran	BT-A, Dysport^®^ analogon
	CosmeTox^®^	Transdermal	USA	BT-A, creme
	BTXA^®^	Intas	India	BT-A, Botox^®^ analogon
	Botogenie	BioMed	India	BT-A, Botox^®^ analogon
	EB-001	Bonti/Allergan	USA	BT-E
	MCL005	Malvern Cosmeceuticals	UK	BT-A, topic gel
	ANT-1207	Anterios/Allergan	USA	BT-A, lotion

From: Dressler 2021

For all BT type A drugs, the duration of action is in the order of 12 weeks. In injections into exocrine glands, it may be longer. For several years, it has been proposed to develop BT drugs with longer or shorter duration of action. It is, however, not quite clear, whether a longer duration of action would benefit patients with chronic disorders usually requiring regular follow-ups anyway to coordinate and adjust the therapeutic setting. A prolonged duration of action may also be a problem, as it would also prolong the duration of adverse effects. A potential use for short duration drugs is even harder to imagine. Modified duration of action will most likely be achieved by modifications of the drug’s BT component or by exploring the human duration of action of non-A BT types.

Traditionally, potency testing during manufacturing is performed by mouse lethality assays. With large numbers of mice being sacrificed every year for this process, it has long been a public demanded to change this practise by applying cell-based assays (Adler et al. 2010). So far, only AbbVie-Allergan and Merz Pharmaceuticals are using cell-based assays. As both are patent-protected, all new BT manufacturers will have to develop own cell-based assays or to license them from those companies.

Potential drug development strategies include exploring recombinant manufacturing processes and development of hybrid BT’s using isolated BT components for targeting or intracellular SNARE protein modulation.

## Outlook

BT therapy has already been proven highly effective and safe for many indications in various medical fields. In the future, we will certainly see further improvements in its treatment algorithms, as we have seen them in the past. Most likely, we will also see a continuous expansion of indications. An expansion of BT into new indication areas, such as depression and arthritis, is still unclear. New drug developments will continue to focus on generating onabotulinumtoxinA and incobotulinumtoxnA analogues. AbobotulinumtoxinA will more and more fall behind, as its idiosyncratic potency labeling prevents comparison with other BT drugs. Whether there is an unmet need for BT drugs with prolonged or reduced duration of action, is unclear. Addition of proprietary proteins does not seem to be an option for this purpose. Liquid BT preparations have not been a relevant selling point in the past and are unlikely to be one in the future—except, maybe, in esthetic medicine.

The biggest challenge, we feel, is the problem is to bring BT therapy to those who need it. In most indications, this market penetration is insufficient, meaning that large numbers of patients, which would benefit from BT therapy, are not reached. Reasons are lack of awareness, education and teaching on one side and therapy costs on the other side. Obviously, both aspects are contrarily correlated with each other. Current high-price business models do not seem to be able to cut this Gordian knot. Therefore, other business models are urgently needed. They might use a biosimilar registration approach or they might be developed in countries where business models are based on different cost structures.
